# A Case Report of Dextrocardia with Situs Inversus: A Rare Condition and Its Clinical Importance

**DOI:** 10.1155/2024/2435938

**Published:** 2024-04-04

**Authors:** Girma Deshimo, Haile Abebe, Getiye Damtew, Enguday Demeke, Seife Feleke

**Affiliations:** ^1^Department of Internal Medicine, Asrat Woldeyes Health Science Campus, Debre Berhan University, Debre Berhan, Ethiopia; ^2^Division of Radiology, Department of Internal Medicine, Asrat Woldeyes Health Science Campus, Debre Berhan University, Debre Berhan, Ethiopia; ^3^Department of Surgery, Asrat Woldeyes Health Science Campus, Debre Berhan University, Debre Berhan, Ethiopia; ^4^School of Public Health, Asrat Woldeyes Health Science Campus, Debre Berhan University, Debre Berhan, Ethiopia

## Abstract

Situs inversus totalis (SIT) is a rare medical condition characterized by a complete mirror-image reversal of the normal positioning of the internal organs. Aristotle initially described situs inversus in animals, while Fabricius first characterized it in humans. Although the specific cause is uncertain, there is evidence of autosomal recessive and X-linked inheritance. Before seeking treatment for an unrelated ailment, many people with SIT are unaware of their distinct anatomy, as in our case. The presented case is a 30-year-old female patient from Central Ethiopia, presented to Hakim Gizaw Teaching Hospital outpatient department of medicine with the complaint of right-sided anterior chest pain for five days. Clinically, the apical beat was heard in the right 5th intercostal space. After undergoing an electrocardiogram (ECG), echocardiogram, chest X-ray, and abdominal ultrasound, she was diagnosed with situs inversus totalis. The clinical implications of SIT encompass challenges in diagnosis and procedures, potential congenital cardiac abnormalities, considerations for organ transplantation, and clinical decision-making, particularly in emergency scenarios. *Key Clinical Messages*. This case highlights the clinical implications of dextrocardia with situs inversus totalis, emphasizing the importance of awareness for accurate diagnosis, procedural challenges, and informed clinical decision-making. Understanding this rare condition is crucial for providing appropriate medical care and to navigate potential complications in affected individuals.

## 1. Introduction

SIT is a rare congenital condition characterized by a complete mirror-image reversal of the normal positioning of the internal organs. Although the precise cause is unknown, many families have experienced different inheritance patterns [1–3]. Situs inversus may be the only anomaly present, or it may be part of a syndrome that includes several other disorders [4–6].

Despite its rarity, understanding this anomaly is crucial due to its unique challenges in clinical diagnosis, management, and treatment due to its atypical presentation and potential for misinterpretation on imaging studies. Due to the uncommon occurrence of situs inversus totalis and the limited understanding of the clinical implications of dextrocardia with situs inversus, there is a critical need to document and analyze individual cases to improve recognition, diagnostic accuracy, and patient outcomes. Therefore, this case report aims to contribute to the existing knowledge base by presenting a detailed clinical description of dextrocardia with situs inversus, emphasizing the rarity of this condition and its clinical implications.

Our case study focused on a patient from Ethiopia who was incidentally diagnosed with situs inversus totalis during an assessment for chest pain.

## 2. Case Presentation

A 30-year-old woman from Sheno, Central Ethiopia, presented to the outpatient department of medicine at Hakim Gizaw Teaching Hospital with five days of right-sided chest pain. The pain was dull aching in character, intermittent with mild to moderate in severity and located anteriorly. It had no radiation to other site, no relieving or aggravating factors identified. There were no other associated symptoms, such as palpitations, shortness of breath, epigastric pain, fever, or headache. She had no history of recurrent upper or lower respiratory tract infections. She had no similar history in the past and had no history of diagnosed chronic illness like hypertension, diabetes mellitus, or chronic lung diseases. This was her first time visit to healthcare facilities.

She looked healthy and had normal vital signs during the physical assessment. Pertinent findings upon physical examination were mild tenderness below the right breast, and S1 and S2 heart sounds were heard well in the right 5^th^ intercostal space. Heart sound auscultation in the right chest was performed, after feeling cardiac pulse while palpating for chest tenderness.

**Figure 1:**
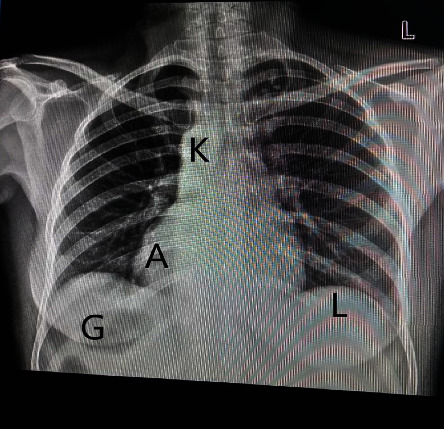
A posteroanterior chest X-ray reveals the cardiac apex (A) and aortic knuckle (K) pointing to the right, the gastric bubble (G) in the right upper quadrant, and the liver (L) shadow in the left.

**Figure 2:**
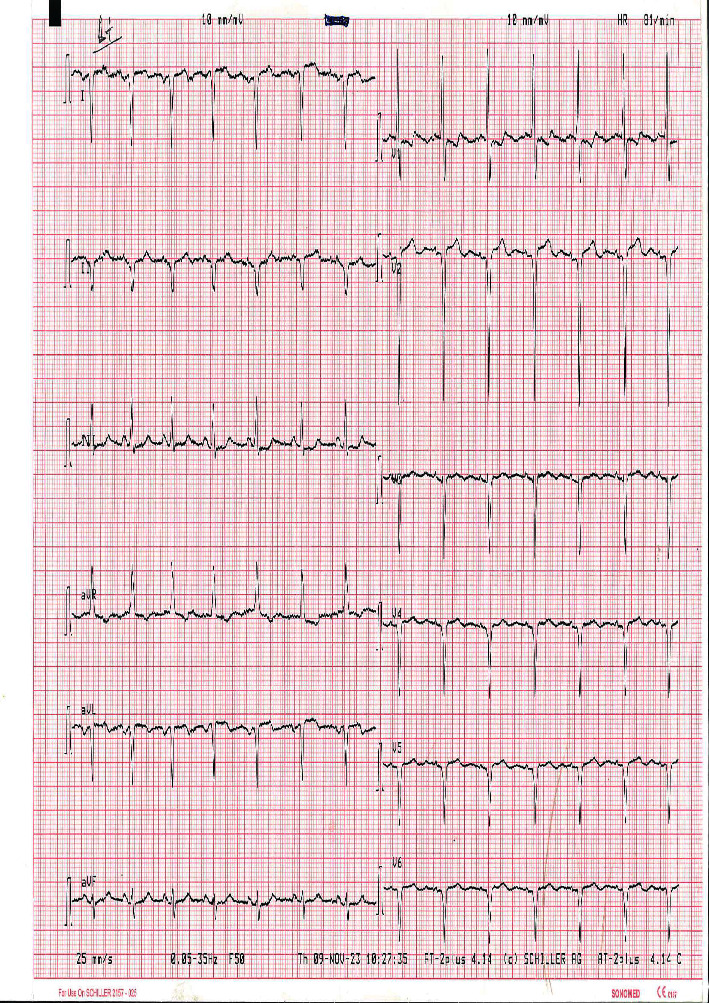
The ECG demonstrating right-axis deviation of the P wave and QRS complex in lead (I) with a negative QRS complex and inverted P and T waves, a positive QRS complex in lead aVR, and absent R-wave progression in precordial leads.

**Figure 3:**
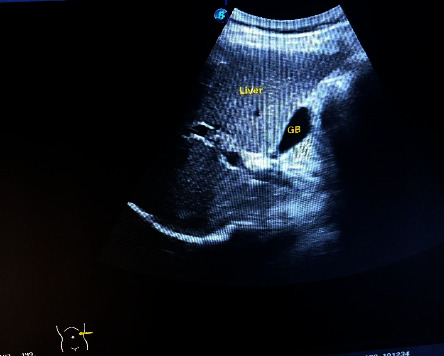
An abdominal ultrasound of the left upper quadrant reveals the gallbladder (GB) and liver on the left side, as opposed to their typical anatomical location.

**Figure 4:**
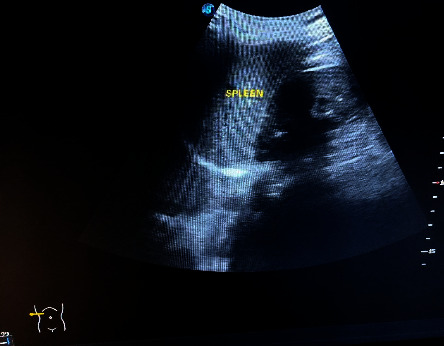
A right-sided spleen is shown in an ultrasonography image of the right hypochondrium.

**Figure 5:**
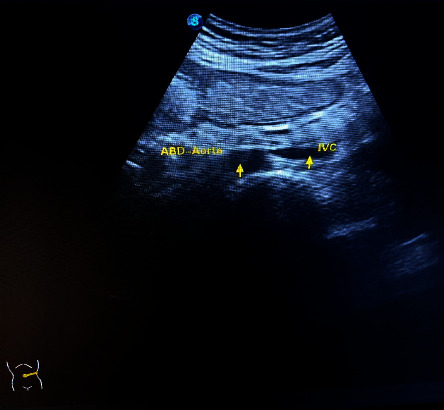
Ultrasound of periumbilical and epigastric region showing anatomical reversal of abdominal aorta and inferior vena cava (IVC).

On investigations: A posteroanterior view of the chest X-ray reveals dextrocardia: a left-sided hepatic shadow, gastric bubble, and the aortic arch on the right side (Figure 1). Additionally, dextrocardia was suggested by the ECG results (Figure 2). Complete blood count and blood sugar were within the normal range. An abdominal ultrasonography revealed situs inversus in the abdomen, with the gallbladder and liver visible at the left upper quadrant (Figure 3), and the spleen was located on the right side (Figure 4). On the left and right sides, respectively, the inferior vena cava and abdominal aorta were visible (Figure 5). In terms of normal anatomy, this is the complete opposite.

After clinical evaluation and investigations, there were no noteworthy results for the chest discomfort and costochondritis was considered in a SIT. The patient was counseled and reassured. Clear explanation was given to her about the anatomical findings. The patient started on acetaminophen and appointed in two weeks. The pain had completely resolved and currently doing well.

## 3. Discussion

SIT is a rare congenital condition characterized by a complete mirror-image reversal of the normal positioning of the internal organs. The typical or normal arrangement of organs, where the stomach and spleen are on the left side of the abdomen, the liver and gallbladder are on the right, and the heart is on the left side of the thorax, is referred to as situs solitus. Partial or complete situs inversus are both possible. Situs inversus with dextrocardia is another name for SIT. It is distinguished by having the liver on the left side of the abdomen, the stomach and spleen on the right, and the heart on the right side of the thorax. The right lung has two lobes, and the left lung has three. The condition known as situs inversus incompletus, or situs inversus with levocardia, is when the heart remains on the left side of the thorax, but the abdominal organs are positioned exactly opposite to where they should be [7, 8].

Fabricius first explained the concept of situs inversus in humans, while Aristotle first explained it in animals. Dextrocardia was initially identified in 1643 by Marco Severino. In 1888, Küchenmeister became the first to physically examine four cases of living individuals and describe the findings with drawings. Vehsemeyer is credited with being the first to show the transposition of the viscera using an X-ray in 1897. Since then, imaging has been the preferred method for clarifying anatomy [9, 10]. The entire reversal arrangement of the abdominal and thoracic organs in situs inversus was described by Matthew Baillie [11].

Situs inversus is a rare congenital abnormality, occurring in approximately 0.001 to 0.01 of the population. It is inherited in an autosomal recessive manner. Situs inversus is the outcome of the viscera and organs rotating in the other direction during the embryo's organogenesis [12–15]. A series of signal molecules and genes coordinate to establish laterality early in development. Szenker-Ravi et al. discovered a set of genes that encode extracellular proteins that directly contribute to the establishment of the left–right axis in animal species with cilia in left–right organizers. Defects in these processes generate heterotaxy, the aberrant development and arrangement of organs across the left–right axis, which can range from full inversion of symmetry to selective organ misarrangement [16, 17].

Many persons who have situs inversus totalis are not aware of their unique anatomy until they go to the physician for anything unrelated. Our case presented with right-sided anterior chest pain.

Chest pain in a patient with situs inversus can present unique challenges in diagnosis and management. Situs inversus itself is not typically associated with chest pain, so it is important to thoroughly investigate the cause of the pain in this context. Given the unusual anatomy of situs inversus patients, healthcare providers should be aware that the location of the pain may be perceived differently due to the reversed positioning of the organs. This can make diagnosing the underlying cause more complex and may require additional imaging or diagnostic tests to accurately identify the source of the pain [18–20]. Furthermore, situs inversus can also be associated with other anatomical variations, such as abnormalities in the positioning of the great vessels or the presence of additional congenital anomalies [21]. These factors can contribute to a differential diagnosis that differs from that of a patient with normal anatomy. Additionally, it is important to consider the possibility of atypical presentations of common conditions in situs inversus patients. For example, conditions, such as heart disease or gastrointestinal issues, may present differently in these patients due to the altered anatomy, so a high level of clinical suspicion and thorough evaluation are crucial. Given the complexity of managing chest pain in a patient with situs inversus, a multidisciplinary approach involving specialists in cardiology, gastroenterology, and radiology may be necessary to fully evaluate and address the underlying cause. Close monitoring and follow-up are essential to ensure that any treatment plan is tailored to the patient's unique anatomical considerations and medical history. Overall, the presence of situs inversus in a patient presenting with chest pain adds an additional layer of complexity to the diagnostic process. Healthcare providers should be prepared to approach the case with a comprehensive understanding of the patient's unique anatomy and potential associated conditions to provide optimal care and management. We evaluated our patient to look for underlying causes for chest pain, but we found no major problems.

Careful physical examinations and imaging investigations are critical to the diagnosis of such patients. Checking for right-sided heart sounds is not a common practice unless there is an indication such as palpable pulsations on the side. Instead, the lack of a left-sided apical beat may raise the possibility of pericardial effusion. However, these patients will not exhibit other pericardial effusion symptoms, such as signs of right-sided heart failure. A variety of factors may contribute to the dullness on the left side and the tympanic in the upper right quadrant of the abdomen, which are clues found during the abdominal examination.

This highlights how crucial imaging is to the diagnosis of SIT. Imaging is important for the diagnosis of associated disorders as well. Traditional imaging modalities, such as ultrasonography or plain film X-ray, are typically the primary choice for diagnostic imaging. Advanced imaging modalities, such as computed tomography (CT) and magnetic resonance imaging (MRI), can be employed to evaluate precise anatomical details and any abnormal findings. Prenatal MRI of the fetus can even provide a complete description of situs abnormalities long before birth. Congenital cardiovascular diseases are frequently discovered in situs inversus, emphasizing the importance of advanced thoracic and abdominal imaging with CT and/or MRI. Combining CT or MRI imaging with fusion techniques can provide a more comprehensive understanding of anatomy that may not be clearly visible on functional imaging [22–27]. The specific imaging modalities used to assess SIT in our case include X-ray, ultrasound, and echocardiography. On a chest X-ray, the heart shadow appeared on the right side of the chest, while the liver shadow was seen on the left side. The positioning of the aortic arch was also reversed. This striking reversal of organ positions on the chest X-ray provided a strong clue to the presence of situs inversus totalis and aid in diagnosis. Abdominal ultrasound further confirmed the diagnosis of situs inversus totalis by demonstrating the mirror-image arrangement of abdominal organs. Dextrocardia was also demonstrated by ECG. Healthcare providers should be familiar with the unique ECG findings associated with SIT to ensure accurate interpretation and appropriate clinical management. Echocardiography was critical in analyzing cardiac anatomy, function, and any potential anomalies that may have arisen as a result of the heart's inverted configuration; in our case, no cardiac abnormalities were discovered. While imaging findings are essential for diagnosing situs inversus totalis, clinical correlation and a thorough evaluation of the patient's symptoms, medical history, and physical examination findings are also crucial.

While it may not cause any significant health problems on its own, it can have clinical importance in certain situations. Situs inversus totalis can make medical diagnoses and procedures more challenging. Healthcare professionals may need to consider the reversed anatomy when interpreting imaging studies, such as X-rays, ultrasounds, or computed tomography scans. This condition can also complicate surgical procedures as surgeons need to adapt their approach accordingly [9, 28–31].

When dextrocardia is present in situs inversus, there is a 3–5% chance of additional congenital cardiac disorders, typically involving the transposition of the great vessels [32]. Kartagener syndrome, which comprises situs inversus totalis, sinusitis, and bronchiectasis, is seen in patients with primary ciliary dyskinesia. No clinically significant cardiopulmonary symptoms were noted in our patient. There have also been reports of renal agenesis [33] in some situs inversus totalis cases. Identifying and managing these cardiopulmonary conditions may require specialized care and interventions.

Organ transplantation in people with SIT presents unique challenges due to the mirror-image reversal of their organs. Surgeons must carefully assess the changed anatomy using modern imaging investigations and have particular surgical expertise to negotiate the challenges of transplant procedures in these patients. Posttransplant care and surveillance are critical for SIT people, as they may experience a higher risk of complications that need rapid intervention [34, 35]. In circumstances where multi-organ transplants are required, coordinating numerous surgeries adds complexity to the treatment plan [36, 37]. Continued research and developments in transplant medicine are required to improve outcomes for SIT patients through innovative surgical procedures and individualized treatment plans. Addressing these concerns can assist to improve the care and treatment outcomes of SIT patients receiving organ transplants.

Because situs inversus totalis is inherited in an autosomal recessive fashion, a child cannot have the condition unless both parents have the mutated gene. For those with situs inversus totalis and their families, genetic counseling may be advised to help them comprehend the likelihood of passing the condition on to future generations [10]. Genetic counseling for patients with SIT and their families can provide valuable information about the condition, assess the risk of inheritance, offer emotional support, assist with family planning decisions, help develop personalized medical management plans, and connect them with resources and support networks. By addressing these aspects, genetic counseling empowers individuals with SIT and their families to better understand the condition, make informed healthcare decisions, and navigate the challenges associated with living with SIT. Our case did not have any known family history of related conditions.

In emergency situations, where quick decision-making is essential, healthcare providers must be aware of SIT. This knowledge is essential for accurate diagnosis and treatment planning to prevent errors and ensure timely interventions [8, 38, 39]. Understanding a patient's unique anatomy due to situs inversus totalis helps anticipate potential challenges and variations in symptoms, leading to improved patient outcomes. Although costochondritis usually resolves on its own, it needs to be distinguished from other, more serious sources of chest pain.

## 4. Conclusion

Situs inversus totalis is uncommon condition in which the body's internal organs are mirrored from their normal locations. The exact cause is still unknown. Situs inversus is often undiagnosed unless it is incidentally discovered during investigations for other medical condition. To arrive at a diagnosis, it is crucial to thoroughly and systematically assess patients. Imaging is essential in the diagnosis of SIT. Physicians should be alerted to search for further associated abnormalities when they find one congenital anomaly. Correct diagnosis is crucial for interpretation of future symptoms and other diagnostic procedures. The clinical implications of SIT encompass challenges in diagnosis and procedures, potential congenital cardiac abnormalities, considerations for organ transplantation, and clinical decision-making, particularly in emergency scenarios. Individuals with SIT should be informed about their condition so that they could inform healthcare providers they might encounter in their subsequent life to ensure appropriate medical care and avoid potential complications.

## Data Availability

The data used to support the findings of this study are available from the corresponding author upon reasonable request.

## Abbreviations

CT: Computed tomography
ECG: Electrocardiogram
MRI: Magnetic resonance imaging
SIT: Situs inversus totalis.
